# Automatic sequence identification in multicentric prostate multiparametric MRI datasets for clinical machine-learning

**DOI:** 10.1186/s13244-025-01938-2

**Published:** 2025-03-27

**Authors:** José Guilherme de Almeida, Ana Sofia Castro Verde, Carlos Bilreiro, Inês Santiago, Joana Ip, Manolis Tsiknakis, Kostas Marias, Daniele Regge, Celso Matos, Nickolas Papanikolaou, ProCAncer-I, José Guilherme de Almeida, Ana Sofia Castro Verde, Manolis Tsiknakis, Kostas Marias, Daniele Regge, Nickolas Papanikolaou

**Affiliations:** 1 Champalimaud Foundation, Lisbon, Portugal; 2 Champalimaud Clinical Center, Lisbon, Portugal; 3 FORTH, Heraklion, Greece; 4 Department of Electrical and Computer Engineering, Hellenic Mediterranean University, Heraklion, Greece; 5 Computational BioMedicine Laboratory, FORTH, Heraklion, Greece; 6 Department of Radiology, Candiolo Cancer Institute, FPO-IRCCS, Candiolo, Italy; 7 Department of Surgical Sciences, University of Turin, Turin, Italy; 8 Department of Radiology, Royal Marsden Hospital, Sutton, UK

**Keywords:** Prostate, Prostatic Neoplasms, Multiparametric magnetic resonance imaging, Data curation, Supervised machine learning

## Abstract

**Objectives:**

To present an accurate machine-learning (ML) method and knowledge-based heuristics for automatic sequence-type identification in multi-centric multiparametric MRI (mpMRI) datasets for prostate cancer (PCa) ML.

**Methods:**

Retrospective prostate mpMRI studies were classified into 5 series types—T2-weighted (T2W), diffusion-weighted images (DWI), apparent diffusion coefficients (ADC), dynamic contrast-enhanced (DCE) and other series types (others). Metadata was processed for all series and two models were trained (XGBoost after custom categorical tokenization and CatBoost with raw categorical data) using 5-fold cross-validation (CV) with different data fractions for learning curve analyses. For validation, two test sets—hold-out test set and temporal split—were used. A leave-one-group-out (LOGO) CV analysis was performed with centres as groups to understand the effect of dataset-specific data.

**Results:**

4045 studies (31,053 series) and 1004 studies (7891 series) from 11 centres were used to train and test series identification models, respectively. Test F1-scores were consistently above 0.95 (CatBoost) and 0.97 (XGBoost). Learning curves demonstrate learning saturation, while temporal validation shows model remain capable of correctly identifying all T2W/DWI/ADC triplets. However, optimal performance requires centre-specific data—controlling for model and used feature sets when comparing CV with LOGOCV, F1-score dropped for T2W, DCE and others (−0.146, −0.181 and −0.179, respectively), with larger performance decreases for CatBoost (−0.265). Finally, we delineate heuristics to assist researchers in series classification for PCa mpMRI datasets.

**Conclusions:**

Automatic series-type identification is feasible and can enable automated data curation. However, dataset-specific data should be included to achieve optimal performance.

**Critical relevance statement:**

Organising large collections of data is time-consuming but necessary to train clinical machine-learning models. To address this, we outline and validate an automatic series identification method that can facilitate this process. Finally, we outline a set of metadata-based heuristics that can be used to further automate series-type identification.

**Key Points:**

- Multi-centric prostate MRI studies were used for sequence annotation model training.
- Automatic sequence annotation requires few instances and generalises temporally.
- Sequence annotation, necessary for clinical AI model training, can be performed automatically.

**Graphical Abstract:**

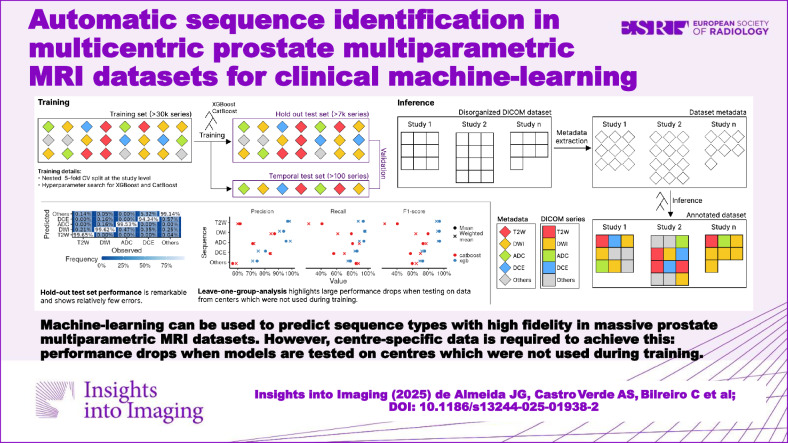

## Introduction

Clinical machine-learning (ML) with radiological data is a growing field [1–4] with clear necessities regarding dataset organisation—there needs to be interoperability between different data sources to allow for meaningful annotations, and series types have to be annotated and identified [5]. At the present moment, plentiful hospitals and clinical centres have access to internal picture archiving and communication systems (PACS) with significant amounts of data, but there are concrete technical and human challenges standing in the way of making such data operational for the training of clinical ML models [6].

A specific problem standing in the way of this operationalisation is the organisation of prostate multiparametric MRI datasets (composed of T2-weighted, diffusion-weighted, apparent diffusion coefficient (ADC) and dynamic contrast-enhanced images [7]) into specific data types while excluding accessory/nonmandatory sequences. Some previous works have focused on this task in the classification of whole-body CT series types [8] or to identify specific brain imaging contrasts [9, 10]. These approaches use deep-learning-based approaches, which we avoid here. Instead, we focus on using the metadata available in Digital imaging and communications in medicine (DICOM) files to classify sequence types as this is more realistic in terms of the typical computational resources available in different clinical centres. Indeed, our approach is similar to that described by Liang et al for brain MRI [11]. Their investigation showed tremendous potential for metadata-based sequence identification, but the relatively small sample size and the absence of testing on data from an external institution hinder the robustness of this work.

Here, we present a sequence-type classification framework based on DICOM metadata to automatically curate large amounts of data. Using a large multi-centric dataset, we also study how performance drops when these methods are applied to data from centres absent from the training data. Finally, we offer a small set of heuristics that can help in curating large prostate mpMRI datasets. This workflow was developed in the context of ProCAncer-i^1^, a Europe-based consortium focused on collecting and developing clinical ML models for prostate mpMRI.

## Methods

**Fig. 1:**
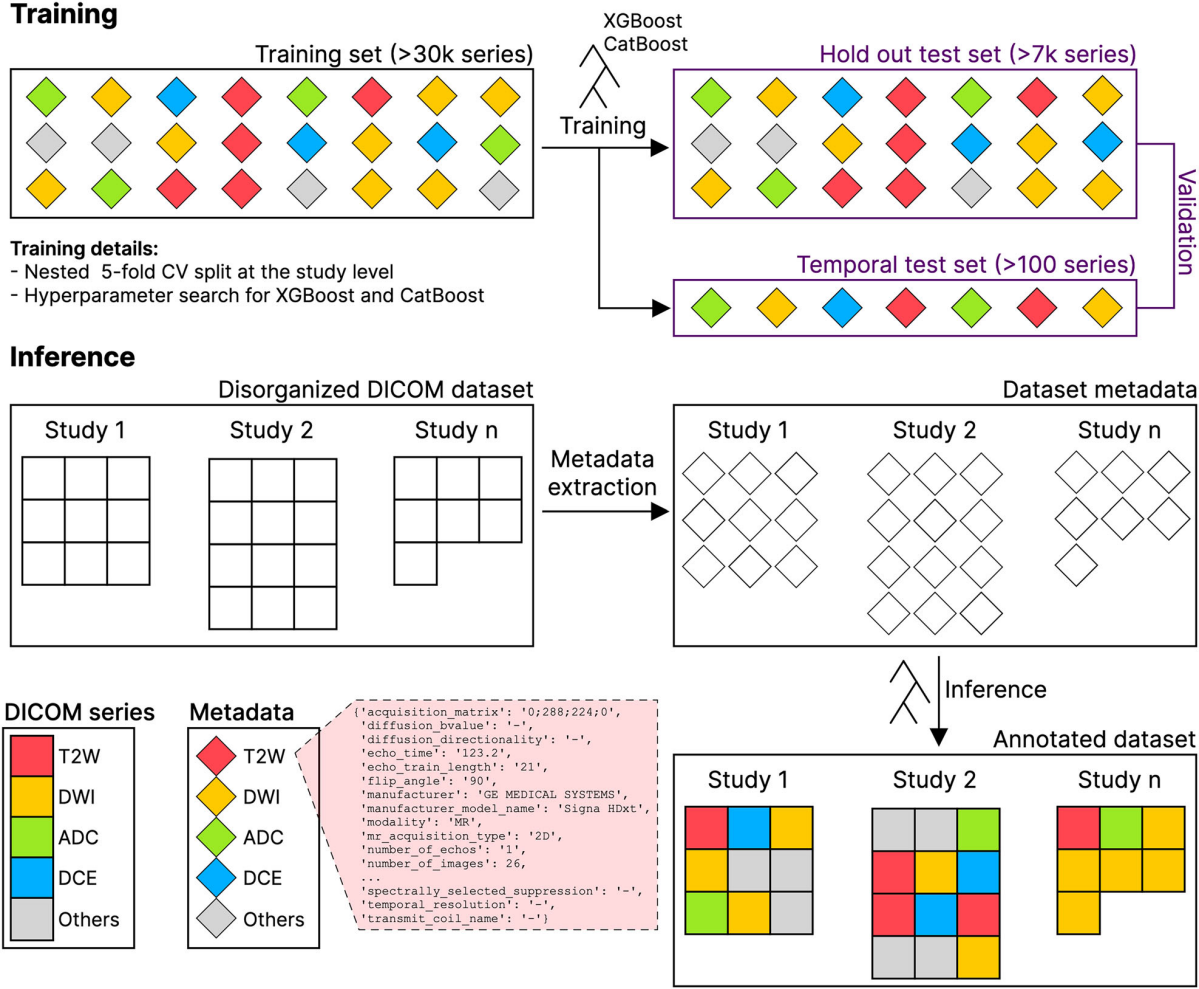
DICOM series-type classification from metadata. Schematic representation delineating the adopted workflow during this work. Top (Training): XGBoost and CatBoost models were trained on a large dataset of annotated DICOM metadata-series type pairs and validated on both a hold-out test set and a temporal test set. Bottom (Inference): the best-performing model is applied to new DICOM data with no series-type annotations after metadata extraction. Each colour corresponds to a series type (T2W, DWI, ADC, DCE, Others) and shapes correspond to DICOM files (squares) or metadata (slanted squares)

We illustrate our training and inference workflows in Fig. 1. Shortly, a large dataset of DICOM metadata-series type pairs was used to train XGBoost and CatBoost models using 5-fold nested cross-validation, during which hyperparameters were tuned. Validation was performed using both a holdout and temporal test set. Inference on new data (DICOM series) follows a two-step process—(i) DICOM metadata extraction and (ii) classification using the best-performing model.

### Dataset

A total of 4045 retrospective prostate mpMRI studies acquired prior to March 31st 2022 with 31,053 series from ProstateNet^2^ were used to train machine-learning models (Table 1). Each series was annotated as one of the following: T2W (T2-weighted), DWI (diffusion-weighted image), ADC (apparent diffusion coefficient), DCE (dynamic contrast enhancement) and others (comprehending any other modality which is typically not used specifically for diagnosis or machine-learning applications). These studies were retrieved from 11 distinct centres (Table 2). For testing, 1004 studies with 7891 series were retrieved from the same centres and annotated (Table 1). Informed consent was waived for retrospective data collection across the different centres.

**Table 1:** Dataset size for training and testing stratified by series type

Series type	Train series count	Test series count
ADC	5031	1276
DCE	2954	865
DWI	7478	1860
T2	5714	1441
Others	9875	2448
Total	31,053	7891

**Table 2:** Number of studies from each centre

Centre	Country	Train studies count	Test studies count
Radboud UMC	Netherlands	1811	458
FPO	Italy	624	162
Champalimaud Foundation	Portugal	566	116
National Cancer Institute	Lithuania	377	97
QUIRONSALUD	Spain	348	79
IDIBGI	Spain	186	58
UNIPI	Italy	89	19
JCC	Portugal	20	7
HULAFE	Spain	13	6
RMH	UK	8	2
HACETTEPE	Turkey	3	0

### Automatic curation

#### Metadata extraction

Specific metadata fields were extracted from each series using pydicom, a Python package for DICOM data handling. Whenever more than one unique value was present for each field, these were processed into a single string value containing all unique values separated by spaces. To harmonise data without removing important information, we clean each metadata field with the following data sanitisation protocol:

1. Dates were removed from series descriptions using a regular expression substitution (i.e. [0-9]+[/-:][0-9]+[/-:][0-9]+ were replaced with an empty sequence);
2. Missing values were replaced with -;
3. A set of non-space characters (|, -, ;,,,(,), _, :)were replaced with space characters;
4. Spaces were squeezed (if more than one consecutive space was present, this was replaced with a single space).

Given that each series has an arbitrary number of DICOM files (slices), the unique values for each series were identified and each metadata field in a given series was characterised as a single value using the space-separated set of unique values for each series.

#### Machine-learning-assisted annotation

Two ML models were tested, CatBoost and XGBoost, as both methods allow missing data and require little hyperparameter optimisation. CatBoost has the added benefit of requiring no explicit tokenization of strings (this is done internally during training if necessary). String tokenization is a process by which strings of characters are split into sets of relevant elements (substrings). Both models were optimised in a machine with a graphics processing unit card (NVIDIA GeForce RTX 3090), 8 cores and 256GB of RAM to predict one of 5 distinct classes—T2W, DWI, ADC, DCE and “others”.

XGBoost,  unlike CatBoost, requires the explicit tokenization of strings. To do this, we considered string columns as belonging to one of two types—categorical columns (columns containing a mixture of alphanumeric characters) and space-separated numerical columns (columns containing a mixture of numeric characters separated by spaces). To handle the former, we tokenize strings using a minimum per-series frequency of 0.01 (each substring should be in at least 1% of the data) and a minimum and maximum n-gram size of 1 and 5 (each string can be split into a maximum of 5 substrings), respectively. Each value in space-separated columns was summarised with 5 distinct features—number of characters and their sum, mean, minimum and maximum values. These operations ensure that string columns are converted into sets of numerical values, directly usable by XGBoost.

Nearly all the retrieved metadata tags were used in training (Supplementary Table 1), and some models were trained using alternative feature sets. In particular, we trained models using 4 feature sets: (i) all features; (ii) without (w/o) series description; (iii) w/o field of view (FOV) and specific absorption rate (SAR); and iv) w/o series description, FOV and SAR. Each model was trained using 5-fold nested cross-validation; during the inner loop, a random set of hyperparameters was picked using random grid search (Supplementary Table 2) for a total of 50 iterations and 5-fold stratified cross-validation was used to estimate performance using the F1-score average across classes; models were retrained using the best-performing parameters and the expected performance was estimated with the outer loop.

To determine the importance of having data from each centre for classification performance, a leave-one-group-out cross-validation analysis (LOGOCV) was performed. For this analysis, each validation fold is composed of all the data for a given centre and the training data is data belonging to any other centre.

#### Learning curve analysis

Learning curves were established to determine the relationship between sample size and performance. Models were trained using 1%, 2%, 5%, 10%, 25%, 50%, 70% and 100% of the data and tested on the same hold-out test set. We use a two-step approach to detect whether more data can lead to performance improvements. This approach first detects whether the rate of performance improvement changes. Then, we confirm that performance is stationary after this point (i.e. performance remains stable as the number of training samples increases). We provide more details in the Supplementary Methods.

### Temporal validation

We used a random set of 102 temporal studies (collected between March 31st 2022 and March 1st 2024) from 4 centres (Quirónsalud, Spain; National Cancer Institute, Lithuania; Royal Marsden Hospital, UK; Hospital La Fe, Valencia, Spain) to validate our model. For this task, radiologists were asked to identify three series typically utilised in clinical ML model development (axial T2W, DWI, ADC; other series types were not identified). This validation was performed to assess whether an expected performance drop was observable with time as this provides a better understanding of whether these models should be updated after their implementation.

### Statistical analysis

To evaluate machine-learning model performance we focused on three metrics—F1-score, recall and precision—as well as on confusion matrices. To determine whether statistically significant differences are present in our data, we use the CV performance and apply linear regression testing using the hold-out test set in order to control for the effect of different modelling decisions on the performance. We used a significance threshold of 0.05.

Model training was performed using CatBoost (version 1.2.2) [12], XGBoost (version 2.0.3) [13] and scikit-learn (version 1.4.1.post1) [14] in Python (version 3.11.8). All post-hoc analysis were performed using R (version 4.1.2); breakpoint analysis was performed using the segmented package (version 2.0-3) [15].

### Derivation of heuristics

While predictive methods can perform remarkably well for sequence-type classification, domain knowledge can also be put to good use. More concretely, we observed whether there were recurrent mistakes in prediction outputs caused by a lack of specificity in the classifications themselves (i.e. it may be important to identify specifically the axial T2W, or to discriminate not only DWI but high *b*-value DWI) and that could be easily solved by applying domain knowledge. Through this process, we have identified a set of heuristics, which we outline below in the results section and describe how they can be easily applied.

## Results

### Near-flawless discrimination of series types

**Fig. 2:**
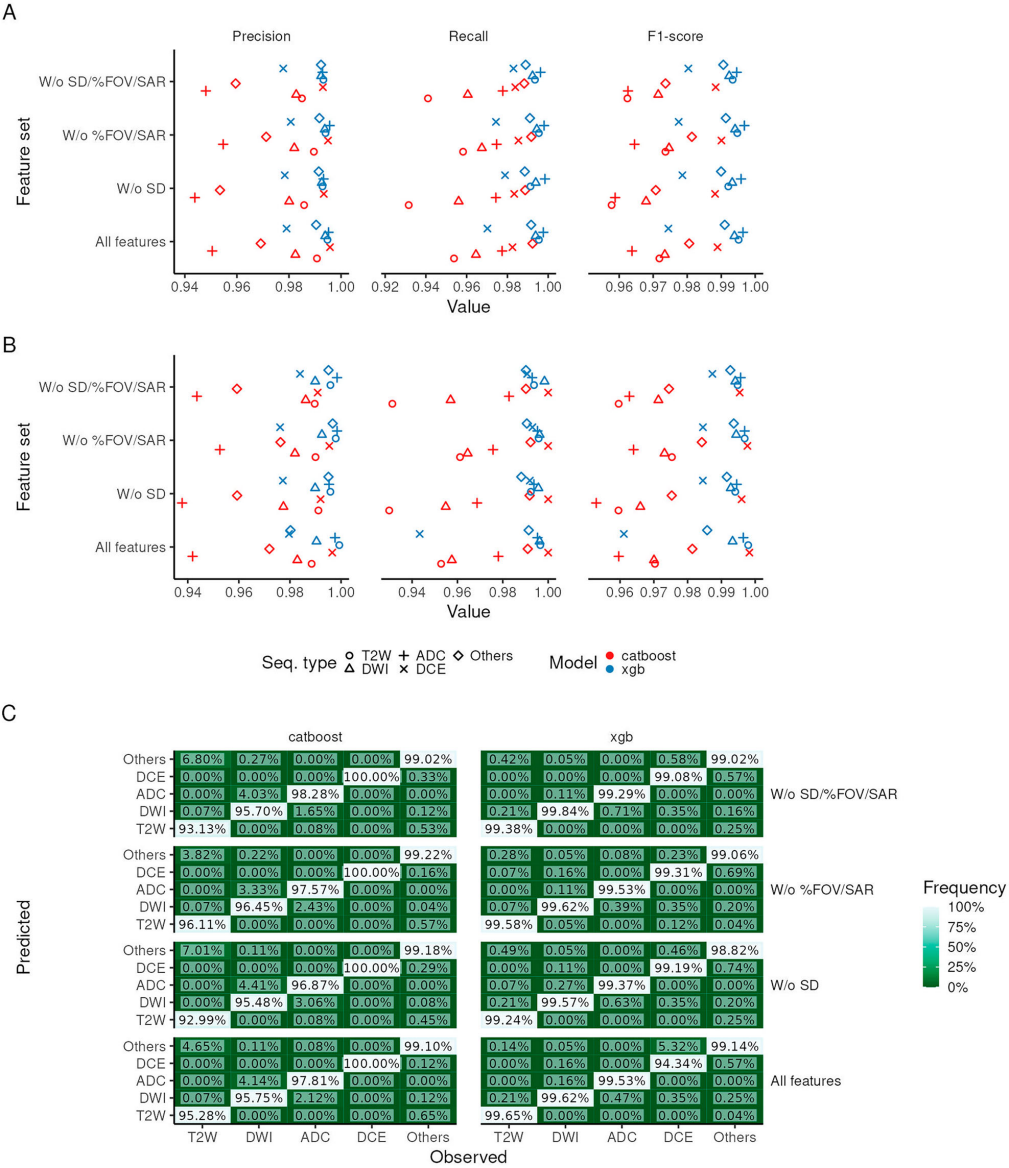
Performance of metadata-based series-type classification models. **A**, **B** Cross-validation and hold-out test set metrics, respectively, stratified by model (colour) and series type (marker shape). **C** Confusion matrix for hold-out test set, stratified by model and series type

While performance features very small errors across all sequence types for both models, some relevant differences are noteworthy:

- **XGBoost outperforms CatBoost**—XGBoost, trained using engineered features (i.e. tokenized string features), outperformed CatBoost, which uses an automated combination of ordered target encoding and combinatorial feature construction (Fig. 2; Table 3; Table 4). While CatBoost performs this (together with other methods) to reduce overfitting, its intended effect is not observed (we initially posited that this may be a consequence of highly variable metadata fields such as series description, but our analysis shows that the performance differences in CatBoost models between including/excluding series description is minimal). This is particularly bad when considering that a large fraction of T2W, typically essential in clinical ML prostate bpMRI models, is oftentimes misclassified as other sequence types (Fig. 2C).

**Table 3:** Average performance for all sequence types and all models stratified by testing split (cross-validation or “CV” and hold-out test set or “Test”)

Exclusion	Model	Split	T2W	DWI	ADC	DCE	Others
W/o %FOV/SAR	CatBoost	CV	97.4% (0.7%)	97.5% (0.6%)	96.5% (0.8%)	**99.0% (0.9%)**	98.1% (0.3%)
W/o SD/%FOV/SAR			96.2% (0.6%)	97.1% (0.8%)	96.3% (1.1%)	98.8% (0.7%)	97.4% (0.5%)
W/o SD			95.8% (1.0%)	96.8% (0.6%)	95.9% (0.7%)	98.8% (1.0%)	97.1% (0.6%)
All features			97.2% (1.0%)	97.3% (0.3%)	96.4% (0.4%)	98.9% (1.3%)	98.1% (0.4%)
W/o %FOV/SAR	XGBoost		**99.5% (0.3%)**	**99.4% (0.4%)**	**99.7% (0.2%)**	97.8% (1.4%)	**99.1% (0.4%)**
W/o SD/%FOV/SAR			99.3% (0.2%)	99.2% (0.3%)	99.5% (0.3%)	98.0% (0.8%)	**99.1% (0.3%)**
W/o SD			99.2% (0.4%)	99.3% (0.3%)	99.6% (0.1%)	97.9% (0.6%)	99.0% (0.4%)
All features			**99.5% (0.4%)**	**99.4% (0.3%)**	99.6% (0.3%)	97.4% (0.9%)	**99.1% (0.4%)**
W/o %FOV/SAR	CatBoost	Test	97.2% (0.4%)	97.2% (0.5%)	96.2% (0.7%)	**99.5% (0.2%)**	98.1% (0.3%)
W/o SD/%FOV/SAR			95.8% (0.4%)	96.8% (0.6%)	96.0% (0.7%)	98.9% (1.0%)	97.1% (0.5%)
W/o SD			95.4% (0.7%)	96.4% (0.3%)	95.3% (0.4%)	98.9% (1.0%)	96.9% (0.4%)
All features			96.8% (0.7%)	96.9% (0.1%)	95.9% (0.1%)	99.2% (1.1%)	97.8% (0.3%)
W/o %FOV/SAR	XGBoost		99.6% (0.1%)	**99.4% (0.2%)**	**99.6% (0.1%)**	97.8% (1.5%)	**99.2% (0.5%)**
W/o SD/%FOV/SAR			99.4% (0.1%)	99.3% (0.0%)	99.5% (0.1%)	98.4% (0.5%)	**99.2% (0.0%)**
W/o SD			99.3% (0.1%)	99.3% (0.1%)	99.4% (0.1%)	97.3% (1.9%)	98.8% (0.5%)
All features			**99.7% (0.1%)**	99.3% (0.1%)	**99.6% (0.0%)**	96.9% (1.2%)	98.8% (0.5%)

Numbers in parentheses correspond to the standard deviation of each estimate rounded to the second decimal place. Standard deviations were calculated using the performances for individual folds. The highest performance for each class at each stage (CV, Test) is highlighted in bold

**Table 4:** Coefficient values, their associated standard error (Std. error) and one-way *t*-tests for a linear model where the hold-out test set F1-score is the dependent variable and categorical factors corresponding to model, feature sets and sequence types were the independent variables

Parameter	Coefficient	Std. error	*t*-value	*p*-value
Model				
CatBoost	0.968	0.00555	174	< 0.0001*
XGboost	0.985	0.00555	177	< 0.0001*
Feature sets (vs. standard)				
W/o SD	−0.0007	0.00524	−0.134	0.895
W/o %FOV/SAR	0.00464	0.00524	0.886	0.382
W/o SD/%FOV/SAR	0.00139	0.00524	0.265	0.793
Sequence type (vs. ADC)				
T2W	0.032	0.00585	0.547	0.588
DWI	0.00403	0.00585	0.688	0.497
DCE	0.0102	0.00585	1.75	0.09
Others	0.00702	0.00585	1.2	0.24

*corresponds to statistically significant variables at a significance threshold of 0.05
- **Including all features leads to larger performance drops for DCE identification in XGBoost**—however, these drops in performance are relatively small (Fig. 2; Table 3) and are unlikely to lead to mis-curated datasets for clinical ML as these typically require only T2W, DWI and ADC and the largest amounts of misclassified instances are DCE being misclassified as other sequence types or vice-versa.

### Learning saturation indicates near-optimal model performance

Models may require more data to achieve optimal performance. To understand this relationship between training dataset size and performance, we trained models with increasing training data fractions and evaluated them on the same hold-out test data.

**Fig. 3:**
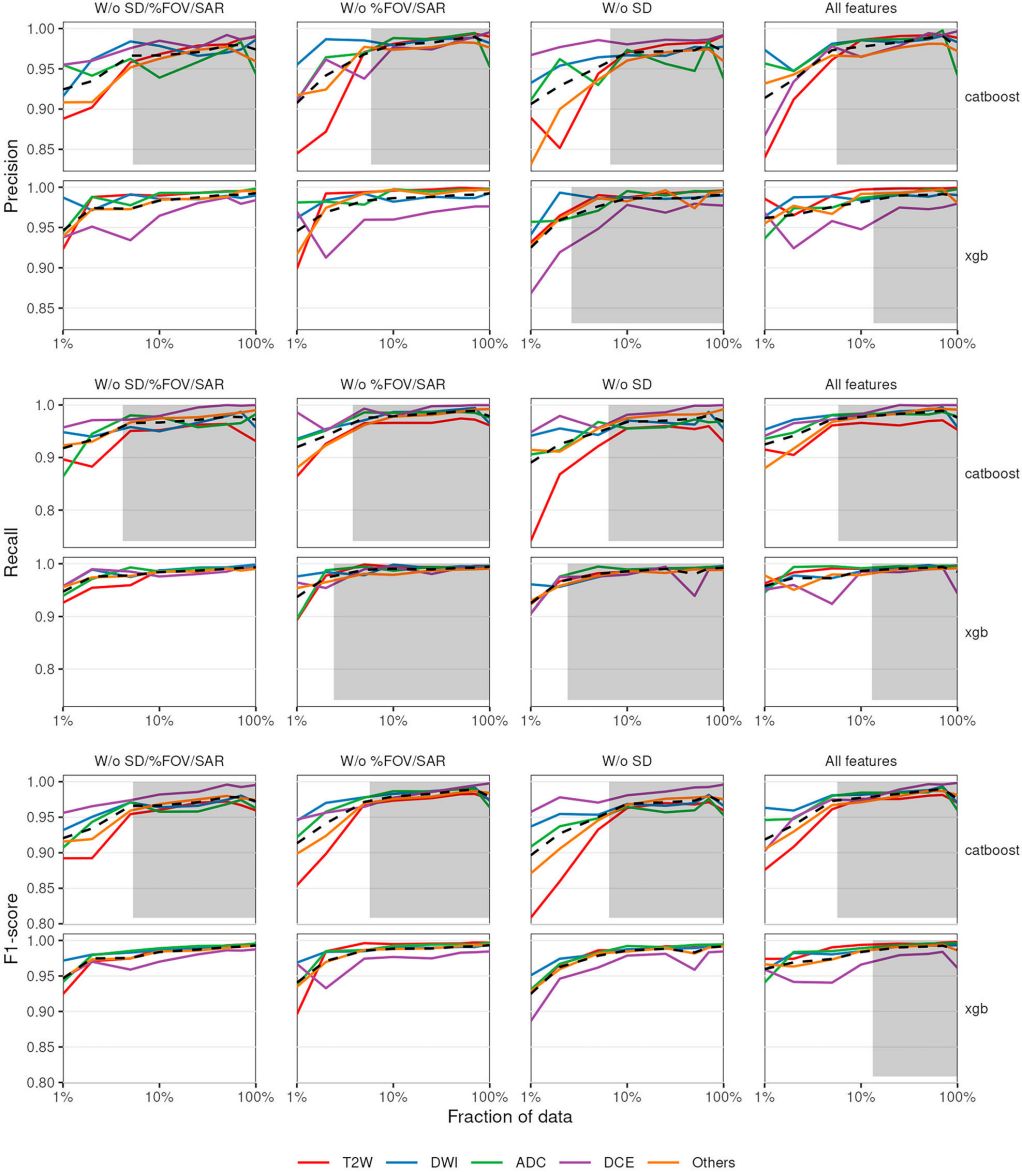
Learning curves for series-type classification models for the hold-out test set. The *x*-axis represents the fraction of data used during training and the *y*-axis represents the value for each metric. Plots are stratified by sequence type (colour). Black lines represent the average performance at each fraction. Shaded grey regions correspond to constant performance

We show that performance stabilises for most models when 2–10% of the training data is used, corresponding to approximately 600–3000 cases (Fig. 3). When this is not the case—precision for XGBoost models w/o SD/%FOV/SAR and w/o %FOV/SAR, recall for XGBoost models without SD/%FOV/SAR and F1-score for XGBoost models w/o SD/%FOV/SAR, %FOV/SAR and SD—this is likely due to inconsistent DCE identification performance. In other words, performance is relatively stable in most cases after an acceptable amount of data is available for the model, implying that optimal performance requires amounts of data smaller than those which were initially annotated.

### Series type discrimination requires dataset-specific cases

The generalisation of these models lies not only on their hold-out test set performance, but also on how they perform when data from a centre is not collected during training. LOGOCV performance shows consistent drops in performance when models are tested on data from centres which were not present during training (Fig. 4A).

However, CatBoost models suffer a considerably larger F1-score drop (Table 5) when compared with XGBoost models. Additionally, different feature sets do not necessarily lead to larger performance drops, while there are very distinct performance drops for different series types. Indeed, ADC and DWI appear to remain relatively easy to identify, while DCE, T2 and other sequences suffer larger performance drops (Fig. 4B). Importantly, for XGBoost models, DCE and T2W are frequently classified as other sequence types. Additionally, ADC is more frequently classified as DWI, particularly in models excluding series description; this is likely due to the fact that radiologists include series-specific information in the series description, and we observed that other tags (i.e. diffusion *b*-value) may be wrongly included in ADC series, making them more similar to DWI.

**Table 5:** Coefficient values, their associated standard error (std. error) and one-way *t*-tests for a linear model where F1-score difference between LOGOCV and CV performance is the dependent variable and categorical factors corresponding to model, feature sets and sequence types were the independent variables

Parameter	Coefficient	Std. error	*t*-value	*p*-value
Model				
CatBoost	−0.265	0.0332	−7.98	< 0.0001*
XGBoost	−0.0337	0.0332	−1.01	0.317
Feature sets (vs. Standard)				
W/o SD	−0.0346	0.0297	−1.16	0.252
W/o %FOV/SAR	0.00623	0.0297	0.21	0.835
W/o SD/%FOV/SAR	−0.0358	0.0297	−1.21	0.235
Sequence type (vs. ADC)				
T2W	−0.146	0.0364	−4.01	0.0003*
DWI	0.0418	0.0364	1.15	0.258
DCE	−0.181	0.0364	−4.98	< 0.0001*
Others	−0.179	0.0364	−4.91	< 0.0001*

*corresponds to statistically significant variables at a significance threshold of 0.05

### Temporal validation highlights consistency

The temporal validation of these models showed their consistency—indeed, the application of the best-performing method to a set of 102 cases obtained after March 31st, 2022 shows that the radiologist-identified triplets of T2W, DWI and ADC are all correctly predicted, and most nuisance series types are correctly identified (73%).

Some nuisance series (series that radiologists did not label as part of the T2W-DWI-ADC triplet) were identified (14%, 12% and 0.8% of nuisance series were identified as T2W, DWI or ADC, respectively). However, we note that all misidentified T2W were correctly identified, but were not adequate for deep learning (sagittal or coronal orientation), had a wider FOV or represented lower quality T2W compared to the T2W selected by the radiologist; similarly, the nuisance series misidentified as DWI series corresponded to lower *b*-value series, whereas the nuisance series classified as ADC series correspond to repeated ADC maps (likely re-calculated based on different sets of *b*-values from DWI series).

### Additional series classification heuristics

Some additional heuristics were identified to further refine sequence annotations for downstream machine-learning analyses. Here, we present and make them available so that they can be of use to other researchers working with large prostate mpMRI data (Fig. 5):

1. Identification of axial plane series in T2W—the identification of an axial plane is of crucial importance as multiple T2W sequences with different orientations may be obtained prior to the completion of the study. To identify the T2W axial sequences (which share the same orientation as DWI and ADC sequences) we started by taking the second column of the direction cosine matrix (which corresponds to the orientation on the *z*-plane) and identifying the index of the largest absolute value. This value—0, 1 or 2—has a direct correspondence to the three identifiable directions—sagittal, coronal and axial, respectively.

**Fig. 4:**
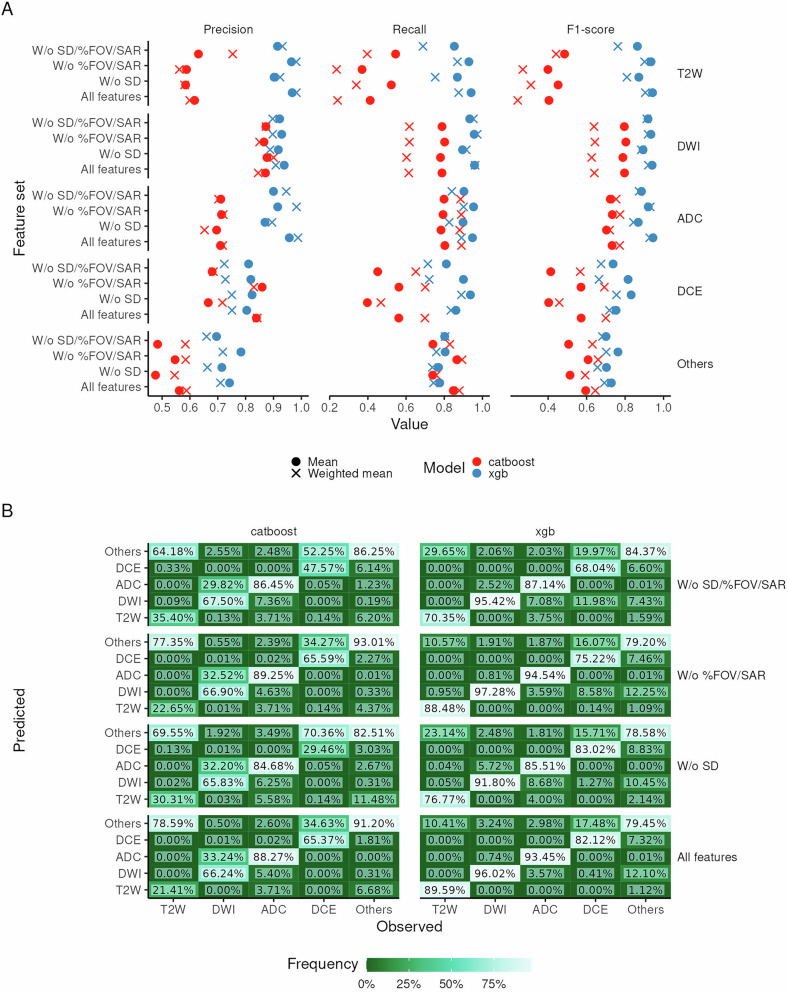
Leave-one-group-out cross-validation performance. **A** Cross-validation metrics stratified by model (colour) and series type; each point represents the mean or weighted mean performance (illustrated with different shapes). **B** Confusion matrix, stratified by model and series type
2. Exclusion of large slice spacings in T2W—from our experience, prostate T2W is typically obtained with approximately 3 mm of between-slice spacing. The exclusion of series with larger spacings is trivial: using the slice spacing tag (0018,0088) in DICOM files, this is easily extractable; we then keep all T2W series which have slice spacing < 4.0 mm.
3. Exclusion of exponential ADC—exponential ADC (eADC) is quantitively different from regular ADC, which was considerably more abundant in ProstateNet. To exclude eADC, we made use of the image type attribute (0008,0008) in ADC DICOM files: this includes “EADC” when images are eADC.
4. Exclusion of synthetic DWI—synthetic DWI (sDWI) are composite images obtained from two regular DWIs at different *b*-values. While they may hold additional diagnostic performance [16], we focused on extracting and working with conventional DWI only. As such, we excluded sDWI by identifying the presence of “synthetic” in the lower-case series description attribute (0008, 103E) of the series predicted as being DWI.
5. *b*-value-based selection of DWI—it is not uncommon for bpMRI/mpMRI studies to have more than one DWI series; however, the most recent PI-RADS guidelines (PI-RADS v2.1) determined that the evaluation of a single high *b*-value (approximately 1400) DWI is sufficient [17]. With this in mind, it is relatively easy to select the highest *b*-value sequence using the (0018, 9087) tag. However, depending on the scanner manufacturer, it is possible that the diffusion *b*-value tag is not specified. For instance, GE or Siemens may use the (0043, 1039) and (0019, 100c) private tags, respectively. With this in mind, automated data curation developers should keep this in mind when selecting high *b*-value DWI for their studies.

## Discussion

In this work, we outline a simple methodology with a comprehensive validation analysis for series-type classification from metadata for prostate MRI. We show that performance is generally good but requires centre-specific data. Finally, we outline some heuristics to further facilitate automatic curation.

The performance achieved here is relatively comparable with that of other studies using either metadata- or deep-learning-based approaches [8–11]; specifically for prostate MRI, a deep-learning-based approach also showed remarkable performance (recall > 90% for external test set performance) [18]. In particular and using larger sample sizes (11,000 series) for brain MRI series-type classification, Jonske and others showed that including a deep-learning module in a metadata-only model led to relatively small performance gains (91.31% vs. 92.71% accuracy for the same approach with and without the deep-learning module, respectively) [19]. This is demonstrative that metadata-based approaches to series type classification perform well and that gains from deep-learning-based methods are limited.

Previous work in this field shows relatively good performance while having smaller dataset sizes [8–11]; others, using considerably larger datasets (> 10,000 series) [19, 20] or much larger datasets than the one used here (600,000 series) [21] showed equally good performance in brain MRI series type classification. This relatively high stability is indicative of robust performance across vastly different training dataset sizes and domains. This is a sensible conclusion—what our learning curve analysis shows is that there is relatively consistent saturation for most models at approximately 10% of the data or even with smaller fractions of data. These aspects are encouraging, as they may motivate centres to implement similar approaches with smaller data collections.

The relatively high heterogeneity in terms of performance when considering the LOGOCV performance may be related to specific factors—while ADC or DWI have indicative tags (i.e. *b*-value or image type) which assist in classification, private tags can make automated metadata extraction more complicated; T2 can have high variability in terms of direction and spacing; and the heterogeneity of the “others” category, with many different types of sequences, may hinder prediction across centres. This has been shown in other studies in brain MRI series-type classification, highlighting a performance drop when models were externally validated [20, 21]. In prostate MRI, this conclusion has also been highlighted for deep-learning-based models—while performing well internally (> 90% for most series types, similar to what is described here), there are consistent performance drops on external test sets [18]. This heterogeneity comes from standard clinical practice—a small study across 23 MRI scanners in London revealed striking amounts of protocol heterogeneity [22], while a study on 44 prostate mpMRI experts showed there were some areas pertaining to mpMRI acquisition, assessment and interpretation where consensus was non-existent or hard to obtain [23]. In other words, while guidelines are available, there is remarkable heterogeneity in terms of protocol and data acquisition, factors which compound with scanner vendor, model or even software versions.

Temporal validation has become a key factor in ML validation as model performance tends to degrade over time [24]; as expected, this also happens in clinical ML models [25, 26], which can be critical for patient-sensitive applications. Here, we offer the first instance of temporal validation for a metadata-based series-type predictor, showing consistent performance, and present a set of knowledge-based heuristics which can help developers address some issues which arose during the multiple validation phases of this work.

**Fig. 5:**
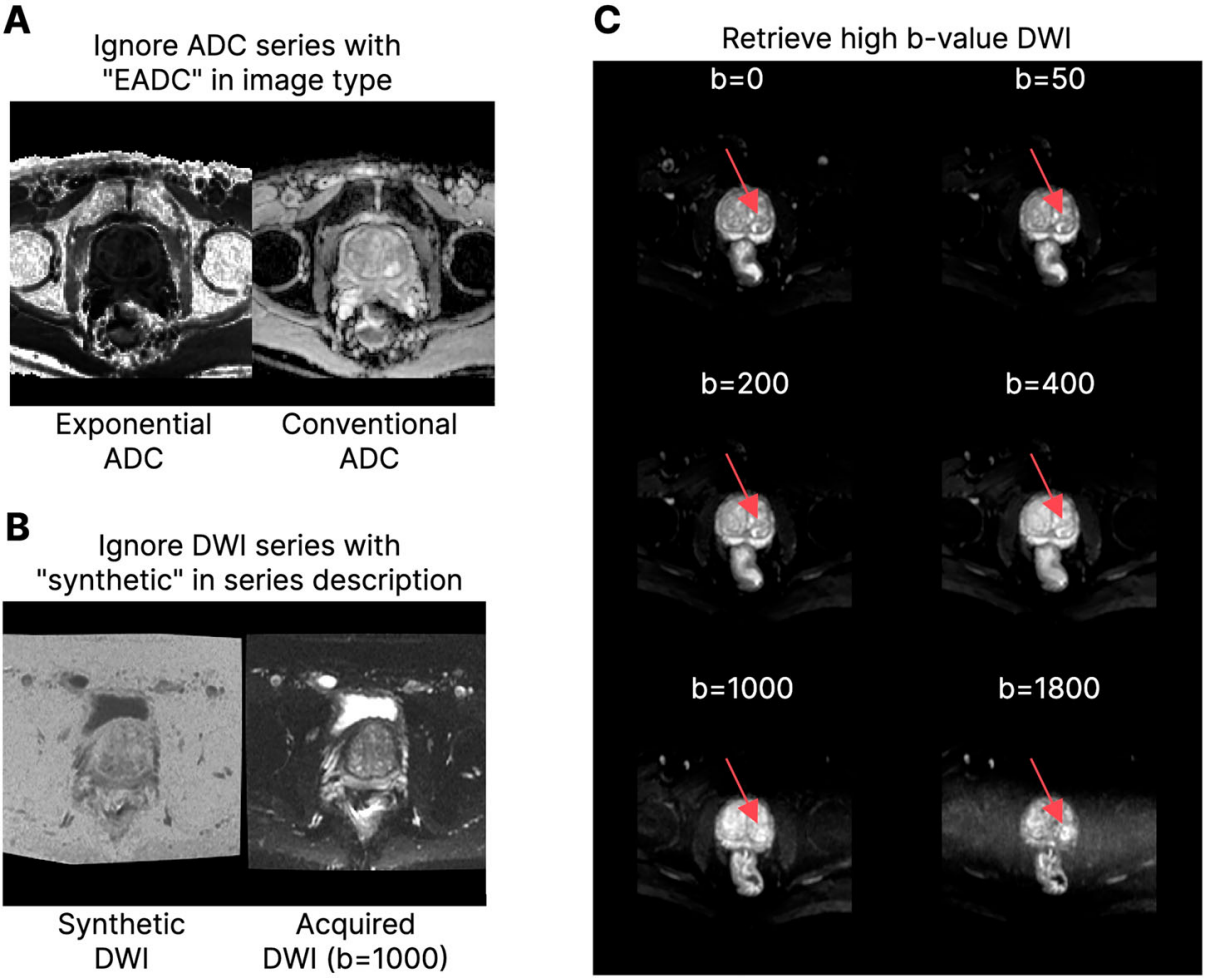
Visual examples of the ambiguities solved by heuristics. **A** Exclusion of exponential ADC (EADC) over conventional ADC. **B** Exclusion of synthetic DWI over acquired DWI. **C** Selection of a sufficiently high *b*-values allowing for better lesion detection (the red arrow represents the location of a potential lesion)

Some limitations can be identified—pixel information was not tested but can potentially improve the relatively poor LOGOCV performance at the cost of increased computational cost. Additionally, we note that there is myriad of ML models which could have been tested here; however, recent benchmarks have shown that tree-based methods (such as CatBoost or XGBoost) consistently outperform other modern approaches [27].

## Conclusion

Using a large dataset of over 30,000 prostate mpMRI series, we have designed a method, which automatically organise large datasets lacking sequence annotations with excellent performance in both retrospective and prospective test sets. However, once we test how these methods generalised to datasets absent from the training data, we observe drops in performance, which hinders the transferral of these methodologies to external medical centres. Finally, we outline a set of useful heuristics that medical informatics teams can use to assist in the automatic curation of large prostate mpMRI datasets.

## Supplementary information

Supplementary Information

Supplementary Information authors name

## Data Availability

The original data cannot be made available as it constitutes sensitive patient information. Processed data (a comma-separated file) containing training and hold-out test data can be made available upon reasonable request. The code available for model training and inference is available at github.com/CCIG-Champalimaud/metadata-classification.

## Abbreviations

ADC: Apparent diffusion coefficient
bpMRI: Biparametric magnetic resonance imaging
DWI: Diffusion-weighted imaging
DICOM: Digital imaging and communications in medicine
DCE: Dynamic contrast enhancement
eADC: Exponential apparent diffusion coefficient
FOV: Field of view
LOGOCV: Leave one-group out cross-validation
ML: Machine-learning
MRI: Magnetic resonance imaging
mpMRI: Multiparametric magnetic resonance imaging
SD: Series description
SAR: Specific absorption rate
T2W: T2-weighted (images)

## Footnotes

1 https://www.procancer-i.eu/.

2 https://prostatenet.eu.
