# Supplementary material for: Automatic sequence identification in multicentric prostate multiparametric MRI datasets for clinical machine-learning
Source: Insights Imaging. 2025 Mar 27;16:75. doi: 10.1186/s13244-025-01938-2 (PMC12187622; doi:10.1186/s13244-025-01938-2)
Supplement: Supplementary file 1 — Supplementary Information [file 13244_2025_1938_MOESM1_ESM.pdf]

Automatic sequence identification in multicentric prostate multiparametric MRI datasets for clinical machine-learning

ELECTRONIC SUPPLEMENTARY MATERIAL

Supplementary methods

As noted in the methods section, the identification of breakpoints was performed using a two-step process. This two step process makes use of a pseudo-Score statistic to identify whether a breakpoint (the point where the slope of a function changes) is statistically significant, and the subsequent determination of stationarity through the use of a linear model. Both steps were performed as follows:

1. Breakpoints (points where the slope changes) were identified using standard linear modelling with unknown breakpoints and breakpoint estimation using data fraction as the independent variable, an intercept term and performance as the dependent variable (precision, recall, F1-score) [1]. To determine whether the presence of a breakpoint was statistically significant a pseudo-Score statistic was used [2]. This allowed the identification of a piecewise (two stage) relationship between data fraction and performance — if learning increases and then saturates after adding data, it is expected that performance increases during a period, after which performance should remain approximately constant.
2. To determine whether performance was stationary after the breakpoint, the subset of performance values at each data fraction after the breakpoint were selected to conduct a simple regression with an intercept term. If there was no evidence for a coefficient different from 0 (i.e. non-significant t-test) we assume that this corresponds to constant/stationary performance. We note here that an non-significant t-test does not necessarily correspond to an absence of effect; as such, stationarity should be interpreted as “an absence of evidence for a non-constant relationship between sample size and performance”.

Supplementary tables

Tag	Tag name	Notes	Used for prediction
0010,0020	Patient ID		
0020,000d	Study UID		
0020,000e	Series UID		
0020,0013	Instance Number		
0018,9087	Diffusion b-value	Retrieved differently for some GE/Siemens scans through private tags (0043,1039 for GE and 0019,100C for Siemens)	X
0018,9075	Diffusion directionality		X
0018,0081	Echo time		X
0018,0091	Echo train length		X
0018,0080	Repetition time		X
0018,1314	Flip angle		X
0018,1312	In-plane phase encoding direction		X
0018,0023	MR acquisition type		X
0018,1310	Acquisition matrix		X
0018,5100	Patient position		
0018,1100	Reconstruction diameter		X
0018,0087	Magnetic field strength		X
0008,0070	Manufacturer		X
0008,1090	Manufacturer model name		X
0018,0089	Number of phase encoding steps		X
0018,0094	Percent phase field of view		X
0018,0095	Pixel bandwidth		X
0018,1250	Receive coil name		X
0018,1251	Transmit coil name		X
0018,1316	SAR		X
0018,0020	Scanning sequence		X
0018,0021	Sequence variants		X
0018,0050	Slice thickness		X
0018,1020	Software versions		X
0020,0110	Temporal resolution		X
0020,0037	Image orientation patient		X
0008,0008	Image type		X
0018,0022	Scan options		X
0028,0004	Photometric interpretation		X
0018,9025	Spectrally selected suppression		X
0018,0082	Inversion time		X
0028,0030	Pixel spacing		X
0018,0086	Number of echos		X
0020,0105	Number of temporal positions		X
0008,0060	Modality		X
0008,103e	Series description		X

Supplementary table 1: Metadata fields retrieved from each series.

Model	Hyperparameter	Values	Optimized
XGBoost	Learning rate	10^{-4} – 0.3	X
	Maximum tree depth	3 – 10	X
	Minimum child weight	1 – 5	X
	Gamma	10^{-4} – 0.1	X
	Sampling method	Gradient based	
	subsample	0.5	
	Tree method	GPU histogram	
CatBoost	Learning rate	0.01 – 0.3	X
	Maximum bin size	100 – 255	X
	Maximum tree depth	4 – 9	X
	L2 leaf regularization	0.1 – 10	X
	Iterations	1000	
	Leaf estimation method	Newton	

Supplementary table 2: Optimized hyperparameters during random grid search for both CatBoost and XGBoost models.

ProCAncer-I Affiliates

José Guilherme de Almeida, Ana Sofia Castro Verde, Manolis Tsiknakis, Kostas Marias, Daniele Regge, and Nickolas Papanikolaou

References

1. Muggeo VMR (2003) Estimating regression models with unknown break-points. Stat Med 22:3055–3071

2. Muggeo VMR (2016) Testing with a nuisance parameter present only under the alternative: a score-based approach with application to segmented modelling. J Stat Comput Simul 86:3059–3067
