# Supplementary material for: Automatic sequence identification in multicentric prostate multiparametric MRI datasets for clinical machine-learning
Source: Insights Imaging. 2025 Mar 27;16:75. doi: 10.1186/s13244-025-01938-2 (PMC12187622; doi:10.1186/s13244-025-01938-2)
Supplement: Supplementary file 2 — Supplementary Information authors name [file 13244_2025_1938_MOESM2_ESM.docx]

| **Name of Contact** | **Institution** |
| --- | --- |
| Manolis Tsiknakis | Institute of Computer Science - Computational BioMedicine Lab |
| Kostas Marias | Institute of Computer Science - Computational BioMedicine Lab |
| Stelios Sfakianakis | Institute of Computer Science - Computational BioMedicine Lab |
| Aikaterini Nikiforaki | Institute of Computer Science - Computational BioMedicine Lab |
| Ioannis Karatzanis | Institute of Computer Science - Computational BioMedicine Lab |
| Varvara Kalokyri | Institute of Computer Science - Computational BioMedicine Lab |
| Eleftherios Trivizakis | Institute of Computer Science - Computational BioMedicine Lab |
| Grigorios Kalliatakis | Institute of Computer Science - Computational BioMedicine Lab |
| Avtantil Dimitriadis | Institute of Computer Science - Computational BioMedicine Lab |
| Dimitris Fotiadis | Institute of Molecular Biology and Biotechnology (FORTH-IMBB/BR) |
| Nikolaos Tachos | Institute of Molecular Biology and Biotechnology (FORTH-IMBB/BR) |
| Eugenia Mylona | Institute of Molecular Biology and Biotechnology (FORTH-IMBB/BR) |
| Dimitris Zaridis | Institute of Molecular Biology and Biotechnology (FORTH-IMBB/BR) |
| Charalampos Kalantzopoulos | Institute of Molecular Biology and Biotechnology (FORTH-IMBB/BR) |
| Nikolaos Papanikolaou | Champalimaud Foundation, Portugal |
| José Guilherme de Almeida | Champalimaud Foundation, Portugal |
| Ana Castro Verde | Champalimaud Foundation, Portugal |
| Ana Carolina Rodrigues | Champalimaud Foundation, Portugal |
| Nuno Rodrigues | Champalimaud Foundation, Portugal |
| Miguel Chambel | Champalimaud Foundation, Portugal |
| Henkjan Huisman | Diagnostic Image Analysis Group, Department of Medical Imaging, Radboud University Medical Center, Nijmegen, The Netherlands |
| Maarten de Rooij | Department of Medical Imaging, Radboud University Medical Center, Nijmegen, The Netherlands |
| Anindo Saha | Diagnostic Image Analysis Group, Department of Medical Imaging, Radboud University Medical Center, Nijmegen, The Netherlands |
| Jasper J. Twilt | Minimally Invasive Image-Guided Intervention Center, Department of Medical Imaging, Radboud University Medical Center, Nijmegen, The Netherlands |
| Jurgen Futterer | Minimally Invasive Image-Guided Intervention Center, Department of Medical Imaging, Radboud University Medical Center, Nijmegen, The Netherlands |
| Luis Martí-Bonmatí | Biomedical Imaging Research Group. Instituto de Investigación Sanitaria La Fe. Valencia, Spain; Medical Imaging Department. Hospital Universitari i Politècnic La Fe. Valencia, Spain. |
| Leonor Cerdá-Alberich | Biomedical Imaging Research Group. Instituto de Investigación Sanitaria La Fe. Valencia, Spain. |
| Gloria Ribas | Biomedical Imaging Research Group. Instituto de Investigación Sanitaria La Fe. Valencia, Spain. |
| Aikaterini Vraka | Biomedical Imaging Research Group. Instituto de Investigación Sanitaria La Fe. Valencia, Spain. |
| Manuel Marfil | Biomedical Imaging Research Group. Instituto de Investigación Sanitaria La Fe. Valencia, Spain. |
| Silvia Navarro | Biomedical Imaging Research Group. Instituto de Investigación Sanitaria La Fe. Valencia, Spain. |
| Asun Torregrosa | Medical Imaging Department. Hospital Universitari i Politècnic La Fe. Valencia, Spain. |
| Claudia Fontenla | Medical Imaging Department. Hospital Universitari i Politècnic La Fe. Valencia, Spain. |
| Emanuele Neri | Academic Radiology, Department of Translational Research, University of Pisa, Via Roma 67, 56126 Pisa, Italy |
| Giacomo Aringhieri | Academic Radiology, Department of Translational Research, University of Pisa, Via Roma 67, 56126 Pisa, Italy |
| Lorenzo Tumminello | Academic Radiology, Department of Translational Research, University of Pisa, Via Roma 67, 56126 Pisa, Italy |
| Vincenzo Mendola | Academic Radiology, Department of Translational Research, University of Pisa, Via Roma 67, 56126 Pisa, Italy |
| Jochen WALZ | Department of Urology, The Institut Paoli-Calmettes, France |
| Konstantin RICHTER | Department of Urology, The Institut Paoli-Calmettes, France |
| Serge BRUNELLE | Department of Radiology, The Institut Paoli-Calmettes, France |
| Matthias Illy | Department of Radiology, The Institut Paoli-Calmettes, France |
| Deniz Akata | Department of Radiology, Hacettepe University Hospitals |
| Mustafa Özmen | Department of Radiology, Hacettepe University Hospitals |
| Ali Devrim Karaosmanoglu | Department of Radiology, Hacettepe University Hospitals |
| Firat Atak | Department of Radiology, Hacettepe University Hospitals |
| Musturay Karcaaltincaba | Department of Radiology, Hacettepe University Hospitals |
| Joan C. Vilanova | Institute of Biomedical Research of Girona Dr. Josep Trueta (IDIBGI), Department of Radiology (IDI), Girona. Spain |
| Carles Biarnes | Institute of Biomedical Research of Girona Dr. Josep Trueta (IDIBGI), Department of Radiology (IDI), Girona. Spain |
| Jurgita Ušinskienė | Diagnostic and interventional radiology department, National cancer institute, Vilnius, Lithuania |
| Rūta Briedienė | Diagnostic and interventional radiology department, National cancer institute, Vilnius, Lithuania |
| Audrius Untanas | Diagnostic and interventional radiology department, National cancer institute, Vilnius, Lithuania |
| Katsaros Vasilis | Agios Savvas Hospital, Greece |
| Georgiou Georgios | Agios Savvas Hospital, Greece |
| Dow-Mu Koh | Radiology & AI Research Hub, The Royal Marsden NHS Foundation Trust, London, UK. Division of Radiotherapy and Imaging, The Institute of Cancer Research, London, UK |
| Robby Emsley | Radiology & AI Research Hub, The Royal Marsden NHS Foundation Trust, London, UK. Division of Radiotherapy and Imaging, The Institute of Cancer Research, London, UK |
| Sharon Vit | Radiology & AI Research Hub, The Royal Marsden NHS Foundation Trust, London, UK. Division of Radiotherapy and Imaging, The Institute of Cancer Research, London, UK |
| Ana Ribeiro | Radiology & AI Research Hub, The Royal Marsden NHS Foundation Trust, London, UK. Division of Radiotherapy and Imaging, The Institute of Cancer Research, London, UK |
| Simon Doran | Radiology & AI Research Hub, The Royal Marsden NHS Foundation Trust, London, UK. Division of Radiotherapy and Imaging, The Institute of Cancer Research, London, UK |
| Tiaan Jacobs | Radiology & AI Research Hub, The Royal Marsden NHS Foundation Trust, London, UK. Division of Radiotherapy and Imaging, The Institute of Cancer Research, London, UK |
| Sheng Yu | Radiology & AI Research Hub, The Royal Marsden NHS Foundation Trust, London, UK. Division of Radiotherapy and Imaging, The Institute of Cancer Research, London, UK |
| Gracián García-Martí | Quirónsalud Hospital / CIBERSAM, ISCIII, Valencia, Spain |
| Daniele Regge | Candiolo Cancer Institute, FPO-IRCCS, Str. Prov.le 142 km 3.95, 10060 Candiolo, Turin, Italy |
| Valentina Giannini | Candiolo Cancer Institute, FPO-IRCCS, Str. Prov.le 142 km 3.95, 10060 Candiolo, Turin, Italy |
| Simone Mazzetti | Candiolo Cancer Institute, FPO-IRCCS, Str. Prov.le 142 km 3.95, 10060 Candiolo, Turin, Italy |
| Giovanni Cappello | Candiolo Cancer Institute, FPO-IRCCS, Str. Prov.le 142 km 3.95, 10060 Candiolo, Turin, Italy |
| Giovanni Maimone | Candiolo Cancer Institute, FPO-IRCCS, Str. Prov.le 142 km 3.95, 10060 Candiolo, Turin, Italy |
| Valentina Napolitano | Candiolo Cancer Institute, FPO-IRCCS, Str. Prov.le 142 km 3.95, 10060 Candiolo, Turin, Italy |
| Sara Colantonio | Institute of Information Science and Technologies of the National Reserch Council of Italy |
| Maria Antonietta Pascali | Institute of Information Science and Technologies of the National Reserch Council of Italy |
| Eva Pachetti | Institute of Information Science and Technologies of the National Reserch Council of Italy |
| Claudia Caudai | Institute of Information Science and Technologies of the National Reserch Council of Italy |
| Giulio del Corso | Institute of Information Science and Technologies of the National Reserch Council of Italy |
| Danila Germanese | Institute of Information Science and Technologies of the National Reserch Council of Italy |
| Andrea Berti | Institute of Information Science and Technologies of the National Reserch Council of Italy |
| Gianluca Carloni | Institute of Information Science and Technologies of the National Reserch Council of Italy |
| Valentina Colcelli | Institute of applied physics "Nello Carrara" (IFAC) |
| Jayashree Kalpathy-Cramer | Mass General Hospital, Boston MA, USA |
| Christopher Bridge | Mass General Hospital, Boston MA, USA |
| Dagoberto Pulido-Arias | Mass General Hospital, Boston MA, USA |
| Mason Cleveland | Mass General Hospital, Boston MA, USA |
| Jay Patel | Mass General Hospital, Boston MA, USA |
| Joao Correia | Biotronics3D Limited , United Kingdom |
| Walter Hernandez | Biotronics3D Limited , United Kingdom |
| Zoi Giavri | Advantis Medical Imaging Single Member P.C., Athens, Greece |
| Paris Ziogkas | Advantis Medical Imaging Single Member P.C., Athens, Greece |
| Christos Pollalis | Advantis Medical Imaging Single Member P.C., Athens, Greece |
| Dimitrios Agraniotis | Advantis Medical Imaging Single Member P.C., Athens, Greece |
| Ana Jimenez-Pastor | Quantitative Imaging Biomarkers in Medicine, Quibim, Valencia, Spain |
| Jose Munuera Mora | Quantitative Imaging Biomarkers in Medicine, Quibim, Valencia, Spain |
| Nikolaus Forgó | Department of Innovation and Digitalisation in Law, University of Vienna, Austria |
| Clara Saillant | Department of Innovation and Digitalisation in Law, University of Vienna, Austria |
| Theresa Henne | Department of Innovation and Digitalisation in Law, University of Vienna, Austria |
| Rodessa May Marquez | Department of Innovation and Digitalisation in Law, University of Vienna, Austria |
